# Draft Genome Sequences of Thalassobacter Strains 1CONIMAR09 and 16PALIMAR09, Two Members of the Roseobacter Lineage Isolated from Coastal Areas of the Mediterranean Sea around Mallorca Island

**DOI:** 10.1128/genomeA.00041-15

**Published:** 2015-03-05

**Authors:** Maria Mas-Lladó, Joana Maria Piña-Villalonga, Isabel Brunet-Galmés, Balbina Nogales, Rafael Bosch

**Affiliations:** Microbiology Lab, Departament de Biologia, Universitat de les Illes Balears (UIB), Palma de Mallorca, Spain

## Abstract

We report the draft genome sequence of two new members of the Roseobacter lineage, Thalassobacter strains 1CONIMAR09 and 16PALIMAR09, which were isolated from the seawater coast of Mallorca Island. Each genome harbored putative genes for obtaining energy by chemolithotrophy and making aerobic anoxygenic photosynthesis.

## GENOME ANNOUNCEMENT

The Roseobacter lineage (*Rhodobacteraceae* family) is a generalist, diverse, and ubiquitous phylogenetically coherent group and is considered to be a significant component of bacterioplankton (up to 20%) (1, 2). Important physiological traits that can be found in this group are (i) the use of a multitude of organic compounds as a sole carbon and energy source and (ii) the ability to obtain energy by chemolithotrophy and by aerobic anoxygenic photosynthesis (2, 3). Given these features and their ecological relevance, the genomes of an increasing number of Roseobacter isolates have been sequenced.

Two strains of the genus Thalassobacter were isolated from the Mediterranean Sea on Mallorca Island, Spain, in March of 2009 by direct plating on marine broth agar (4). Strain 1CONIMAR09 was isolated from pristine seawater, while strain 16PALIMAR09 was isolated from the seawater of Palma harbor. 16S rRNA gene analysis suggested that both strains affiliate with Thalassobacter stenotrophicus species (99.79% identity with CECT 5294^T^). Currently, only a single genome of Thalassobacter is available (Thalassobacter arenae DSM 19593, accession no. NZ_AONI00000000.1).

Genome sequencing was done using Illumina technology, and the reads were assembled using Newbler version 2.9 (454 Life Sciences). Genome annotation and analysis were done using Prokaryotic Genomes Automatic Annotation Pipeline (PGAAP) at NCBI (http://www.ncbi.nlm.nih.gov/genomes/static/Pipeline.html), the KEGG Automatic Annotation Server (5), JSpecies (6), and the IS-Finder (7) programs.

The results of average nucleotide identity based on BLAST (ANIb) (6) showed that the two strains were members of the same species (99.5% genome identity).

The draft genome of 1CONIMAR09 has 28 contigs and is 3,411,796 bp in length (57-fold coverage). The G+C content is 58.64 mol%. It contains 2,997 coding sequences (CDSs), and at least 40 tRNAs were predicted. The rRNA operon was divided into two different contigs whose fold coverage (141-fold) suggested that there is probably a minimum of two rRNA operons. Genome analysis suggests the presence of at least 19 plausible transposases belonging to 11 different insertion sequence families, as well as 17 integrase-like proteins.

In the case of 16PALIMAR09, the reads were assembled into 39 contigs with a length of 3,531,769 bp (48-fold coverage). The genome has 58.84 mol% G+C content, and it contains 3,345 CDSs and 43 tRNAs. The rRNA operon was coded in one contig, whose coverage (133-fold) also suggested the presence of at least two rRNA operons. Eleven integrase-like proteins and 15 plausible transposases that belonged to eight different insertion sequence families were also predicted.

Putative genes for major metabolic pathways (i.e., glycolysis and citrate cycle) were predicted. Additionally, both genomes contain the *dmdA* gene that is involved in the demethylation pathway of dimethylsulfoniopropionate (DMSP).

In reference to their chemolithotrophic capabilities, both genomes harbored all putative genes for sulfite oxidation (*sox* system) and for carbon monoxide oxidation (two systems of *cox* genes and operons *coxMSL* and *coxSLM*).

Finally, the majority of the genes required for the formation of bacteriochlorophyll-containing photosystems were found. These include *bch* and *crt* genes coding for the bacteriochlorophyll and carotenoid biosynthetic pathways, *puf* and *puc* genes coding for two types of light-harvesting complexes (*pufBA* and *pucBAC*), the reaction center complex (*puhA* and *pufLM*), and regulatory proteins (*ppsR*, *ppaA*, and *tspO*).

### Nucleotide sequence accession numbers.

The whole-genome shotgun projects of strains 1CONIMAR09 and 16PALIMAR09 have been deposited at DDBJ/EMBL/GenBank under the accession numbers JGVS00000000 and JHAK00000000, respectively. Versions described in this paper are JGVS01000000 and JHAK01000000, respectively.
